# Refractory Colitis in Hermansky-Pudlak Syndrome: A Surgical Case Report

**DOI:** 10.7759/cureus.86548

**Published:** 2025-06-22

**Authors:** Miguel Serpa-Irizarry, Derick Rodriguez-Reyes, Ellis D Mejias-Febres, Jean C Lafontaine, Maria Correa, Aura Delgado-Cifuentes

**Affiliations:** 1 General Surgery, University of Puerto Rico, Medical Sciences Campus, San Juan, PRI; 2 School of Medicine, University of Puerto Rico, Medical Sciences Campus, San Juan, PRI; 3 Pathology, University of Puerto Rico, Medical Sciences Campus, San juan, PRI; 4 Pathology and Laboratory Medicine, University of Puerto Rico, Medical Sciences Campus, San Juan, PRI

**Keywords:** granulomatous colitis, hermansky-pudlak syndrome, infliximab-refractory, laparoscopy, low anterior resection, sigmoidectomy, sigmoid stricture

## Abstract

Hermansky-Pudlak syndrome (HPS) is a rare autosomal recessive disorder characterized by oculocutaneous albinism, platelet dysfunction, and, in some subtypes, pulmonary fibrosis and colitis. HPS-associated colitis, particularly subtypes HPS-1 and HPS-4, often mimics Crohn’s disease but exhibits a more refractory course, frequently necessitating immunomodulatory therapy and, in severe cases, surgical intervention. We present the case of a 24-year-old Puerto Rican male with infliximab-resistant HPS-associated colitis who developed a symptomatic sigmoid stricture, requiring laparoscopic low anterior resection with protective loop ileostomy. Preoperative hematologic optimization was essential due to the inherent platelet dysfunction in HPS. Histopathology confirmed chronic active colitis with transmural inflammation, fistulous tract formation, and ceroid deposition. This case underscores the complexity of managing refractory HPS colitis and highlights the role of early recognition and disease monitoring, optimization, and surgical intervention.

## Introduction

Hermansky-Pudlak syndrome (HPS) is a rare autosomal recessive disorder characterized by oculocutaneous albinism (OCA), bleeding diathesis, and, in some cases, pulmonary fibrosis, colitis, and immunodeficiency [1-5]. A distinguishing characteristic of HPS, not observed in other forms of OCA, is the lack of dense bodies in platelets, which leads to an increased tendency for bruising and bleeding. [6] It is genetically heterogeneous, with 11 subtypes caused by mutations in genes affecting lysosome-related organelles, including components of BLOC and AP-3 complexes [1-7]. HPS-1 and HPS-4 are linked to pulmonary fibrosis, while HPS-2 is associated with neutropenia and infections [2,4,6-10]. HPS-3, HPS-5, and HPS-6 typically show milder hypopigmentation, and HPS-9 involves mutations in BLOC1S6 [3,4]. Identifying the subtype is crucial, as it guides prognosis and management, particularly for complications such as pulmonary fibrosis and immunodeficiency [1-6]. HPS is particularly prevalent in Puerto Rico due to a founder mutation in the HPS1 gene; specifically, it is a 16-bp duplication in exon 15, which is associated with HPS-1 [2,3,11]. This region has a high concentration of HPS-1 cases, and patients often present with a range of skin pigmentation and an increased risk of pulmonary fibrosis [3,12]. However, patients with HPS of non-Puerto Rican descent are also found in many other parts of the world [13].

HPS-associated colitis is a well-documented complication, particularly in HPS-1 and HPS-4. The literature indicates that colitis resembling Crohn's disease, often granulomatous in nature, can occur in HPS patients, with a significant number of cases reported among those with HPS-1 and HPS-4 genotypes [4-6]. The colitis in HPS can be severe and may require aggressive treatment, including corticosteroids, immune modulators, and anti-TNFα therapy, similar to treatments used for Crohn's disease [5,6,8]. The presence of colitis in HPS patients is thought to be related to the genetic susceptibility inherent in these subtypes, although the exact pathophysiological mechanisms remain under investigation [6,8]. As such, treatment strategies often parallel those of Crohn's disease, with a primary focus on medical therapy. Surgical intervention is typically reserved for refractory cases or complications, with procedures tailored to the unique manifestations of the disease. We present the case of a Puerto Rican male with a history of HPS and infliximab-resistant colitis, who developed a colonic stricture necessitating surgical management.

## Case presentation

A 24-year-old male with a history of HPS and HPS-associated colitis presented with abdominal discomfort and progressively worsening constipation despite over-the-counter laxatives and stool softeners. His initial diagnosis of HPS-associated colitis was established via colonoscopy in 2016 following a similar history of bowel irregularity and non-specific abdominal symptoms. His medical history was significant for idiopathic pulmonary fibrosis, diagnosed after recurrent asthma-like symptoms. He had no prior surgeries, a non-contributory family history, and a social history notable for cigarette and cannabis use.

Initially, our patient was treated with mesalamine and prednisone for six years and was then transitioned to infliximab therapy (10 mg/kg every four weeks). While infliximab partially controlled his symptoms, surveillance colonoscopies revealed persistent severe inflammation in the sigmoid colon and mild rectal inflammation. A subsequent colonoscopy performed in October 2022 demonstrated a sigmoid stricture approximately 15 cm from the anal verge, which was tattooed for identification. Further examination of the colon was impossible secondary to luminal narrowing caused by the stricture. Due to persistent and progressive symptoms unresponsive to medical therapy and the inability to assess the colon fully, the patient was referred to colorectal surgery.

After discussing the risks versus benefits of surgical management, the patient was scheduled to undergo laparoscopic low anterior resection with loop ileostomy. In preparation for surgery and in addition to mechanical bowel preparation, the hematology service evaluated the patient due to HPS-associated platelet dysfunction. A preoperative regimen of tranexamic acid, desmopressin, and platelet transfusion was recommended to optimize perioperative hemostasis. After the induction of general anesthesia and administration of prophylactic antibiotics, the abdominal cavity was accessed using a Veress needle at Palmer's point, followed by strategic trocar placement under direct vision. Intraoperative findings included a grossly inflamed sigmoid colon extending into the rectum. Mobilization proceeded beyond the tattoo marking to healthy rectal tissue. The splenic flexure was mobilized to ensure a tension-free anastomosis. A 15-cm-long colon segment was removed, and a low rectal anastomosis, 6 cm from the anal verge, was performed using a 25-mm EEA™ circular stapler with Tri-Staple™ technology (Medtronic, Minneapolis, MN), with integrity confirmed by intact tissue rings and a negative air leak test. A protective loop ileostomy was then created by exteriorizing the terminal ileum approximately 20 cm from the ileocecal valve through a right lower quadrant incision. No major bleeding or intraoperative complications occurred. The patient tolerated the procedure well and had an uneventful postoperative recovery, being discharged on postoperative day three.

**Figure 1:**
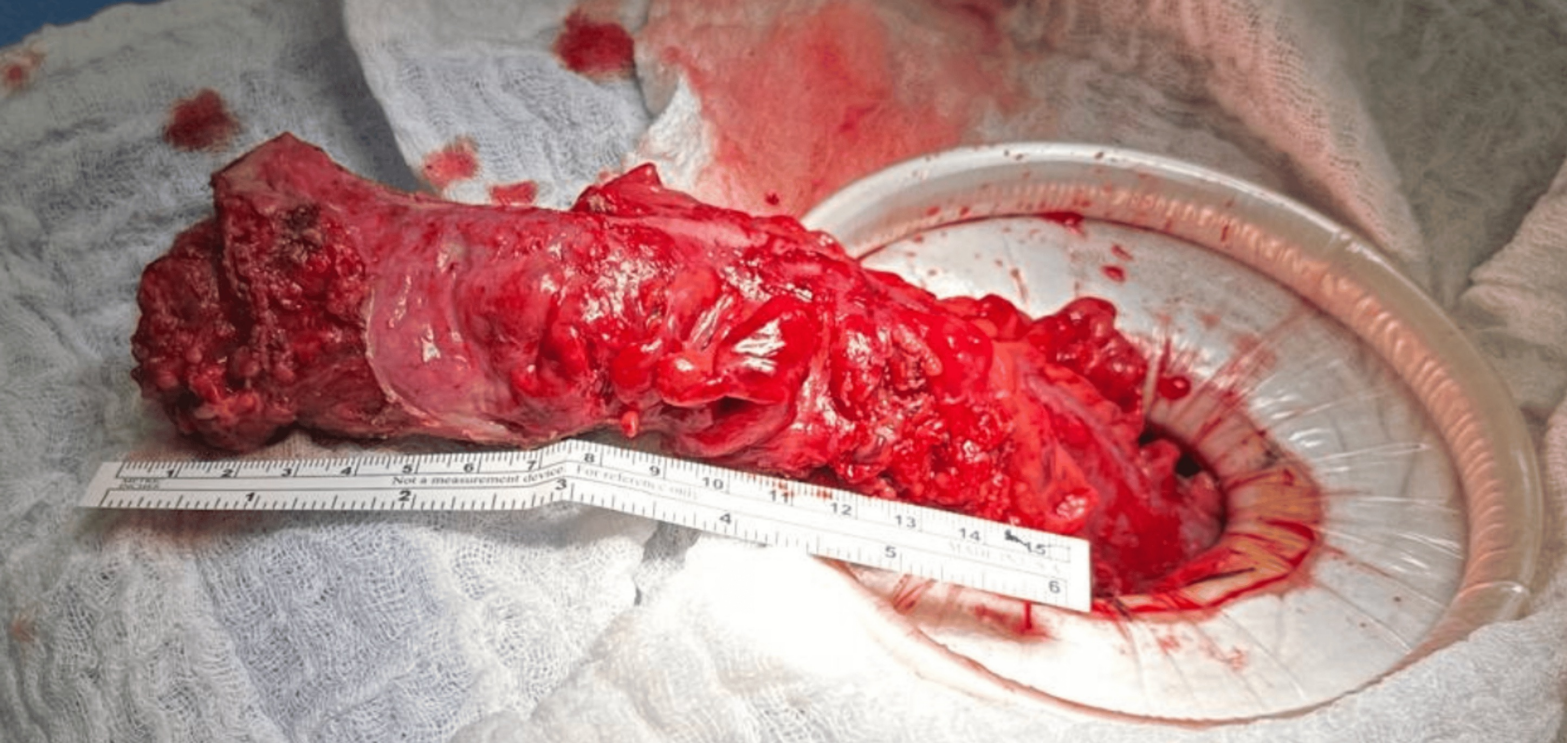
Intraoperative appearance of the sigmoid colon. The sigmoid colon shows severe inflammation, fibrosis, and stricture formation, indicating active severe colitis.

Pathology revealed a segment of the colon with focal serosal fibrous adhesions and abundant pericolonic fat (Figure 1).

**Figure 2:**
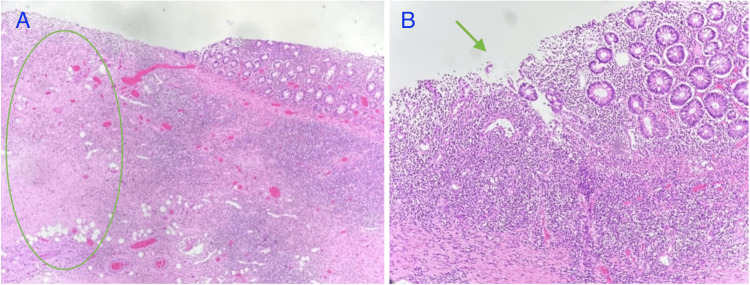
A. Note focal transmural inflammation (oval), H&E, 10x. B. Focal mucosal ulceration (arrow), H&E, 20x.

**Figure 3:**
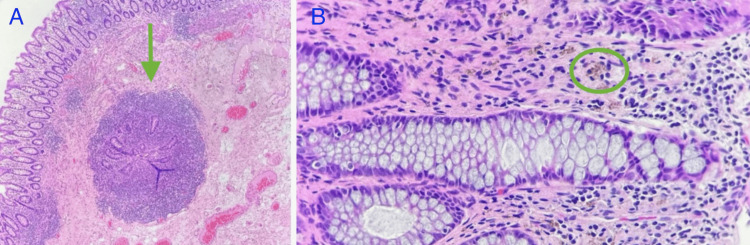
A. Fistulous tract H&E 10x (arrow). B. Fine deposits of yellow-brown pigment consistent with ceroid deposition in lamina propria (oval), H&E, 40x.

The diffusely thickened bowel wall measured up to 0.8 cm, narrowing the lumen significantly, correlating with the clinical findings of obstruction. The mucosa was tan, with irregular folds and focal ulcerations. Margins of resection were grossly uninvolved by disease. The microscopy disclosed chronic active proctocolitis with focal mucosal ulceration, transmural inflammation, fistulous tract formation, focal ceroid deposition, and serosal fibrous adhesions (Figures 2, 3).

Even though granulomatous colitis is a well-documented finding of HPS, no granulomas were identified in this specimen. Immunohistochemistry for CD68, a marker for macrophages was negative, supporting the absence of histiocytic aggregates in granuloma formation.

## Discussion

HPS-associated colitis is a recognized complication of HPS, where it mimics Crohn's disease but often demonstrates greater resistance to standard therapies. There is no consensus as to which should be the standard therapy since their response to classical Crohn’s therapy lacks consistency [14-18]. Previous studies evaluating the efficacy of other agents such as sulfasalazine, mesalazine, corticosteroids, and antibiotics failed to show consistent benefit [13]. Currently, it is most often initially managed with steroids; however, due to high rates of relapse and detrimental systemic consequences, immunologic therapies are now the mainstay of treatment, with infliximab being the most common [9,17-21]. Despite its common use, infliximab has shown variable efficiency in the existing literature, and there are no established guidelines to direct biologic therapy. In this case, infliximab, the cornerstone of treatment for inflammatory bowel disease, failed to control symptoms or prevent disease progression, underscoring the aggressive nature of colitis in some HPS patients and the need for alternative management strategies.

Further evaluation of the surgical pathology showed the characteristic ceroid deposition and confirmed the severity of the disease, including marked luminal narrowing, ulcerations, transmural inflammation, fistulous tracts, and serosal adhesions, which justified surgical management. The margins of resection were grossly uninvolved by disease, and the tattooed portion was effectively removed. Interestingly, our specimen did not show the characteristic granuloma formation associated with HPS colitis. This discrepancy may reflect an underlying spectrum of immune dysregulation rather than a uniform granulomatous etiology as previously thought. As discussed by Miranda et al. in 2024, variability in granuloma formation among HPS patients may relate to differences in underlying BLOC/AP-3 complex mutations, suggesting that the immune mechanisms in HPS colitis differ subtly from Crohn’s disease [17]. In their retrospective review of 27 patients with chronic granulomatous colitis, Alvarez-Downing et al. reported that all of their patients who required surgery had perianal disease [20]. This is congruent with our case since clinically our patient did not show evidence of perianal disease and the pathologic specimen did not show granuloma formation. This finding highlights the complexity of the previously mentioned pathophysiologic mechanisms leading to this disease process and underscores the need for further investigations to elucidate targeted therapies, which, in turn, may avoid the need for surgery.

Surgical planning for HPS patients is complex due to platelet dysfunction inherent to the disorder. In this case, preoperative optimization with tranexamic acid, desmopressin, and platelet transfusion enabled a safe laparoscopic resection with minimal blood loss. Due to the varied presentation of the disease, surgical management is often individualized and targets each complication according to the anatomic location, disease severity, and comorbidities. The chronicity and subacute nature of our patient’s presentation favored a bowel-preserving approach. Reported cases in the literature describe a myriad of procedures including small bowel resection, appendectomy, colostomy, subtotal colectomy, total colectomy, proctocolectomy, and perianal procedures, with segmental colectomy and ileostomy being the most common [15-18,20,21]. Colonic stents may also form part of a conservative approach; however, they are associated with a high complication rate and are usually reserved for palliative cases or as a bridge for surgery in the emergent setting [22]. The decision to perform a limited low anterior resection in our patient highlights the similar principles in Crohn's surgery with the aim of conserving as much bowel as possible in a non-emergent elective surgery by removing the grossly diseased portion alone. Performing a loop ileostomy has been previously described for these high-risk cases with favorable outcomes and was part of a conservative approach. Our rationale was to mitigate postoperative complications in the setting of active disease and significant inflammation in a patient with a low rectal anastomosis and HPS-associated comorbidities including bleeding diathesis, immunodeficiency, and pulmonary fibrosis.

This case adds to the growing literature supporting surgical intervention as a valid, and sometimes necessary, strategy in medically refractory HPS-associated colitis. If possible, a multidisciplinary approach to preoperative optimization and a generally conservative approach is encouraged. While rare, these cases challenge clinicians to recognize that HPS-associated colitis is not merely a variant of Crohn’s disease but a distinct entity with overlapping presentation and varying manifestations. This complexity favors further investigations to elucidate the etiological mechanisms of this disease process and, in turn, develop effective therapeutic modalities.

## Conclusions

This case illustrates the complexity of the presentation and management of HPS-associated colitis, particularly in patients with refractory disease unresponsive to traditional infliximab therapy. Our case also emphasizes the importance of surveillance and early recognition of refractory disease to prevent complications and disease progression. With appropriate preoperative optimization, laparoscopic low anterior resection with protective loop ileostomy provided a safe and effective approach for the management of our patient’s colitis and its complications. Further studies are needed to better understand the pathophysiology of colitis in HPS in order to establish targeted therapies and standardized management protocols for this rare but debilitating disease.
